# Supplementary material for: Pathobiological and Genomic Characterization of a Cold-Adapted Infectious Bronchitis Virus (BP-caKII)
Source: Viruses. 2018 Nov 19;10(11):652. doi: 10.3390/v10110652 (PMC6266813; doi:10.3390/v10110652)
Supplement: Supplementary file 1 [file viruses-10-00652-s001.pdf]

Supplementary Table S1. Amino acid changes during SNU9106 passages through embryonated chicken eggs.

Gene	Amino acid changes [a]				
	E1 (SNU9106)	E5	E10	E15	E20 (BP-caKII)
nsp 7	E34G	D43Y	D43Y	**S4I**, D43Y	**S4I**, D43Y
nsp 12	L28M, L30K, L110F, E123D, D468E, K512Q, **S567N**, K900R	**P214L**, S567N, **P812L**, E832D	**P214L**, S567N, **P812L**, E832D	**P214L**, S567N, **P812L**, E832D	**P214L**, S567N, **R626C**, **P812L**, E832D
nsp 13	N102S, E104D, K206R, **F291S**, I293L, E507D, **P555Q**, **S559N**, **G594D**	S51L, **F291S**, I474V, **T512I**, P555Q, S559N, G594D	S51L, I474V, **T512I**, P555Q, S559N, G594D	S51L, **E168D**, I474V, **T512I**, P555Q, S559N, G594D	S51L, I474V, **T512I**, P555Q, S559N, G594D
nsp 15	**I22M**, **I38V**, A128V, Y143H, T178M, **D202E**, **V249A**, N253S, **I265M**, **I297N**, D301E, T323A	I22M, I38V, V133I, N173S, D202E, V249A, I265M, I297S	I22M, I38V, V133I, N173S, D202E, V249A, I265M, I297S, **K308R**, **S313L**	I22M, I38V, V133I, N173S, D202E, V249A, I265M, I297S	I22M, I38V, V133I, N173S, D202E, V249A, I265M, I297S, K308R, **S313L**
S	**S2L, G3V, L5S, L8I, L12S, V21F, F23S, 24G, 25N ins., H26N, H56Y, G62S, S64P, A79V, S96A, Y116F, L128M, Q131R, N132D, K154E, T200N, N202S, V218A, D226N, D227S, R229K, S252T, K255R, Q256E, R257K, T271A, H278T, E280V, T281S, S284Q, N291S, S292T, I293F, S294H, T295L, K319I, E320K, N322D, L336P, K369R, S393Q, T422I, R445Q, R457S, S482G, E483A, Y484H, G519D,**	S2L, V21D, D22N, F23 del., H24N, V66A, R118M, F273L, S365I, K488E, F697L, S883F, S1006F,	S2L, V21D, D22N, F23 del., H24N, V66A, R118M, F273L, S365I, K488E, F697L, I847F, S879T, S883F, S1006F,	S2L, V21D, D22N, F23del., V66A, R118M, F273L, S365I, K488E, F697L, I847F, S879T, S883F, S1006F,	S2L, V21D, D22N, F23 del., H24N, V66A, R118M, F273L, S365I, K488E, V500L, F697L, I847F, S879T, S883F, S1006F,
E	I3V, K6N, E9D, A57V,	L27F,	L27F, **G23A**	L27F, **G23A**	L27F
M	I26V, I35L, I47F, V55M, I72V, D76N, T77M, L92F, S144A, S223N		**L80F**		**L80F**
N	T7A, S101A, S151G, A179T, I232V, P244L, L298V, R299T, S342P, T343N, S351N, R356K	S109R, K136T, V317L, T343S, N407Y	S109R, K136T, V317L, T343S, N407Y	S109R, K136T, V317L, T343S, N407Y	S109R, K136T, **I204V**, V317L, T343S, N407Y

[a] Amino acid changes were determined by comparison to corresponding amino acid sequences of KM91.
